# Supplementary material for: Pre-Birth Household Challenges Predict Future Child’s School Readiness and Academic Achievement
Source: Children (Basel). 2022 Mar 15;9(3):414. doi: 10.3390/children9030414 (PMC8947585; doi:10.3390/children9030414)
Supplement: Supplementary file 1 [file children-09-00414-s001.zip › Figure_S1.pdf]

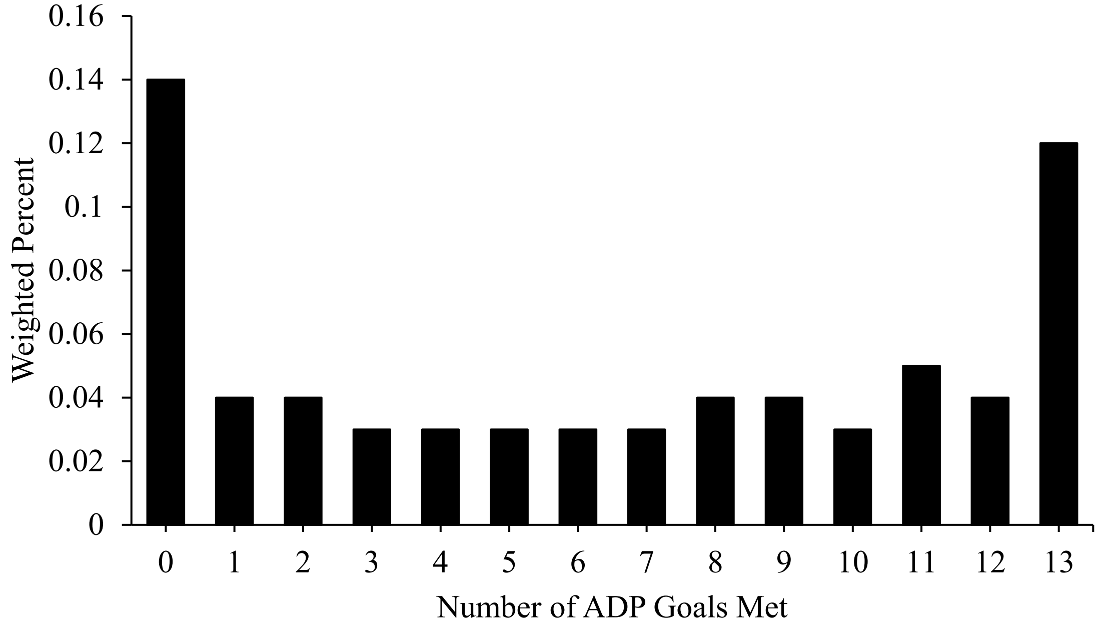


Figure S1. Weighted percent distribution of number of Alaska Developmental Profile (ADP) goals met in population. Meeting at least 11 out of 13 goals is considered to be the developmental gold standard.
