# Supplementary material for: Pre-Birth Household Challenges Predict Future Child’s School Readiness and Academic Achievement
Source: Children (Basel). 2022 Mar 15;9(3):414. doi: 10.3390/children9030414 (PMC8947585; doi:10.3390/children9030414)
Supplement: Supplementary file 1 [file children-09-00414-s001.zip › Table_S1.pdf]

Table S1. Independent Associations of Pre-Birth Household Challenge Components 12 Months Before Birth of 3-Year-Old Child with Child's ADP Goals Met

Pre-Birth Household Challenges		≥ 11* (*N*)	< 11 (*N*)	< 11 Weighted Mean (95% CI)	Risk Ratio (95% CI)
**Moving to a New Address**					
	Yes	221	551	0.70 (0.66, 0.74)	1.01 (0.94, 1.09)
	No	457	1150	0.69 (0.66, 0.71)	Referent
**Someone Close Had Drug or Drinking Issue**					
	Yes	112	362	0.74 (0.69, 0.79)	1.10 (1.02, 1.19)
	No	568	1,336	0.68 (0.65, 0.70)	Referent
**Can't Pay Bills**					
	Yes	114	363	0.75 (0.70, 0.80)	1.11 (1.03, 1.20)
	No	564	1,333	0.67 (0.65, 0.70)	Referent
**Birthing Parent Lost Job**					
	Yes	48	153	0.74 (0.66, 0.82)	1.08 (0.97, 1.21)
	No	627	1,534	0.68 (0.66, 0.71)	Referent
**Partner Lost Job**					
	Yes	55	194	0.79 (0.73, 0.85)	1.17 (1.07, 1.27)
	No	621	1,497	0.68 (0.65, 0.70)	Referent
**Mental Health Check or Treatment**					
	Yes	62	162	0.73 (0.66, 0.80)	1.06 (0.95, 1.17)
	No	618	1,560	0.69 (0.66, 0.71)	Referent
**Homeless**					
	Yes	19	77	0.84 (0.75, 0.93)	1.23 (1.09, 1.38)
	No	661	1,621	0.68 (0.66, 0.71)	Referent
**Birthing Parent or Partner in Jail**					
	Yes	39	114	0.73 (0.64, 0.82)	1.06 (0.93, 1.21)
	No	639	1,580	0.69 (0.66, 0.71)	Referent
**Divorce or Separation**					
	Yes	43	156	0.79 (0.72, 0.86)	1.16 (1.05, 1.28)
	No	636	1,540	0.68 (0.66, 0.70)	Referent
**Death in Family**					
	Yes	105	346	0.74 (0.69, 0.79)	1.09 (1.01, 1.18)
	No	574	1,350	0.68 (0.65, 0.70)	Referent
**Sick Family Member**					
	Yes	121	341	0.72 (0.67, 0.77)	1.06 (0.98, 1.15)
	No	560	1,354	0.68 (0.65, 0.70)	Referent
**Argued with Partner More Than Usual**					
	Yes	130	406	0.74 (0.69, 0.78)	1.09 (1.01, 1.18)
	No	545	1,284	0.68 (0.65, 0.70)	Referent

Table S1 continued.

**Partner Didn't Want Pregnancy**					
	Yes	32	115	0.76 (0.67, 0.85)	1.11 (0.99, 1.26)
	No	644	1,577	0.68 (0.66, 0.71)	Referent
**Intimate Partner Violence**					
	Yes	41	118	0.74 (0.66, 0.82)	1.08 (0.96, 1.22)
	No	636	1575	0.68 (0.66, 0.71)	Referent
**Physical Fight**					
	Yes	9	41	0.71 (0.59, 0.83)	1.03 (0.87, 1.22)
	No	651	1,627	0.69 (0.66, 0.71)	Referent

* Referent category for outcome variable
ADP: Alaska Developmental Profile
CI: Confidence Interval
