# Supplementary material for: Pre-Birth Household Challenges Predict Future Child’s School Readiness and Academic Achievement
Source: Children (Basel). 2022 Mar 15;9(3):414. doi: 10.3390/children9030414 (PMC8947585; doi:10.3390/children9030414)
Supplement: Supplementary file 1 [file children-09-00414-s001.zip › Table_S2.pdf]

Table S2. Independent Associations of Pre-Birth Household Challenge Components 12 Months Before Birth of 3-Year-Old Child with 3rd Grade PEAKS ELA Evaluation Score

Pre-Birth Household Challenges		A/P* (*N*)	BP/FBP (*N*)	BP/FBP Weighted Mean (95% CI)	Risk Ratio (95% CI)
**Moving to a New Address**					
	Yes	128	273	0.64 (0.58, 0.70)	1.01 (0.91, 1.13)
	No	267	594	0.63 (0.59, 0.67)	Referent
**Someone Close Had Drug or Drinking Issue**					
	Yes	53	218	0.76 (0.70, 0.83)	1.26 (1.14, 1.40)
	No	341	648	0.60 (0.57, 0.64)	Referent
**Can't Pay Bills**					
	Yes	62	207	0.75 (0.68, 0.82)	1.24 (1.12, 1.38)
	No	332	654	0.60 (0.57, 0.64)	Referent
**Mom Lost Job**					
	Yes	32	86	0.72 (0.61, 0.83)	1.15 (0.97, 1.35)
	No	362	773	0.63 (0.59, 0.66)	Referent
**Partner Lost Job**					
	Yes	33	105	0.72 (0.63, 0.82)	1.16 (1.01, 1.33)
	No	362	755	0.62 (0.59, 0.66)	Referent
**Mental Health Check or Treatment**					
	Yes	32	87	0.77 (0.68, 0.86)	1.23 (1.09, 1.40)
	No	364	789	0.62 (0.59, 0.66)	Referent
**Homeless**					
	Yes	12	42	0.90 (0.83, 0.97)	1.44 (1.32, 1.58)
	No	383	824	0.62 (0.59, 0.66)	Referent
**Birthing Parent or Partner in Jail**					
	Yes	14	62	0.79 (0.67, 0.92)	1.26 (1.07, 1.49)
	No	380	801	0.63 (0.59, 0.66)	Referent
**Divorce or Separation**					
	Yes	22	91	0.82 (0.73, 0.91)	1.33 (1.17, 1.50)
	No	371	773	0.62 (0.58, 0.65)	Referent
**Death in Family**					
	Yes	50	190	0.74 (0.67, 0.81)	1.20 (1.08, 1.35)
	No	344	674	0.61 (0.58, 0.65)	Referent
**Sick Family Member**					
	Yes	89	173	0.63 (0.56, 0.70)	0.99 (0.88, 1.13)
	No	305	692	0.64 (0.60, 0.67)	Referent
**Argued with Partner More Than Usual**					
	Yes	63	234	0.77 (0.70, 0.83)	1.28 (1.16, 1.42)
	No	329	628	0.60 (0.56, 0.64)	Referent

Table S2 continued.

**Partner Didn't Want Pregnancy**					
	Yes	17	62	0.78 (0.66, 0.90)	1.24 (1.05, 1.46)
	No	375	804	0.63 (0.59, 0.66)	Referent
**Intimate Partner Violence**					
	Yes	11	74	0.90 (0.83, 0.98)	1.46 (1.33, 1.62)
	No	383	789	0.62 (0.58, 0.65)	Referent
**Physical Fight**					
	Yes	6	43	0.84 (0.69, 0.98)	1.33 (1.11, 1.60)
	No	386	823	0.63 (0.59, 0.66)	Referent

* Referent category for outcome variable
PEAKS: Performance Evaluation for Alaska's Schools Reading Assessment
ELA: English language arts
A/P: Score of Advanced or Proficient on PEAKS
BP/FBP: Score of Below Proficient or Far Below Proficient on PEAKS
CI: Confidence Interval
