# Supplementary material for: Pre-Birth Household Challenges Predict Future Child’s School Readiness and Academic Achievement
Source: Children (Basel). 2022 Mar 15;9(3):414. doi: 10.3390/children9030414 (PMC8947585; doi:10.3390/children9030414)
Supplement: Supplementary file 1 [file children-09-00414-s001.zip › Table_S3.pdf]

Table S3. Independent Associations of Pre-Birth Household Challenge Components 12 Months Before Birth of 3-Year-Old Child with Child's Average School Attendance

| Pre-Birth Household Challenges | | ≥ 90%* (*N*) | < 90% (*N*) | < 90% Weighted Mean (95% CI) | Risk Ratio (95% CI) |
|---|---|---|---|---|---|
| **Moving to a New Address** | | | | | |
| | Yes | 644 | 234 | 0.23 90.20, 0.27) | 1.16 (0.98, 1.38) |
| | No | 1,317 | 448 | 0.20 (0.18, 0.22) | Referent |
| **Someone Close Had Drug or Drinking Issue** | | | | | |
| | Yes | 357 | 172 | 0.30 (0.25, 0.34) | 1.54 (1.29, 1.85) |
| | No | 1,604 | 509 | 0.19 (0.17, 0.21) | Referent |
| **Can't Pay Bills** | | | | | |
| | Yes | 397 | 136 | 0.22 (0.18, 0.26) | 1.05 (0.85, 1.29) |
| | No | 1,560 | 543 | 0.21 (0.19, 0.23) | Referent |
| **Mom Lost Job** | | | | | |
| | Yes | 166 | 60 | 0.22 (0.16, 0.29) | 1.06 (0.78, 1.45) |
| | No | 1,781 | 618 | 0.21 (0.19, 0.23) | Referent |
| **Partner Lost Job** | | | | | |
| | Yes | 217 | 61 | 0.20 (0.14, 0.26) | 0.94 (0.70, 1.27) |
| | No | 1,738 | 614 | 0.21 (0.19, 0.23) | Referent |
| **Mental Health Check or Treatment** | | | | | |
| | Yes | 181 | 68 | 0.25 (0.18, 0.32) | 1.18 (0.89, 1.56) |
| | No | 1791 | 624 | 0.21 (0.19, 0.23) | Referent |
| **Homeless** | | | | | |
| | Yes | 73 | 44 | 0.38 (0.27, 0.50) | 1.87 (1.36, 2.56) |
| | No | 1,888 | 636 | 0.20 (0.19, 0.22) | Referent |
| **Birthing Parent or Partner in Jail** | | | | | |
| | Yes | 103 | 56 | 0.33 (0.24, 0.42) | 1.60 (1.21, 2.13) |
| | No | 1,853 | 624 | 0.21 (0.19, 0.22) | Referent |
| **Divorce or Separation** | | | | | |
| | Yes | 155 | 69 | 0.29 (0.22, 0.37) | 1.44 (1.11, 1.88) |
| | No | 1,805 | 610 | 0.20 (0.19, 0.22) | Referent |
| **Death in Family** | | | | | |
| | Yes | 332 | 167 | 0.29 (0.25, 0.34) | 1.51 (1.26, 1.82) |
| | No | 1,627 | 513 | 0.19 (0.18, 0.21) | Referent |
| **Sick Family Member** | | | | | |
| | Yes | 391 | 126 | 0.23 (0.18, 0.27) | 1.09 (0.88, 1.33) |
| | No | 1,569 | 554 | 0.21 (0.19, 0.23) | Referent |
| **Argued with Partner More Than Usual** | | | | | |
| | Yes | 429 | 171 | 0.25 (0.21, 0.29) | 1.27 (1.05, 1.54) |
| | No | 1,523 | 506 | 0.20 (0.18, 0.22) | Referent |

Table S3 continued.

| | | | | | |
|---|---|---|---|---|---|
| **Partner Didn't Want Pregnancy** | | | | | |
| | Yes | 116 | 44 | 0.18 (0.12, 0.24) | 0.86 (0.60, 1.22) |
| | No | 1,840 | 632 | 0.21 (0.19, 0.23) | Referent |
| **Intimate Partner Violence** | | | | | |
| | Yes | 117 | 56 | 0.27 (0.19, 0.35) | 1.29 (0.95, 1.74) |
| | No | 1,836 | 625 | 0.21 (0.19, 0.23) | Referent |
| **Physical Fight** | | | | | |
| | Yes | 70 | 34 | 0.24 (0.15, 0.33) | 1.14 (0.76, 1.71) |
| | No | 1,888 | 647 | 0.21 (0.19, 0.23) | Referent |

* Referent category for outcome variable

CI: Confidence interval
